# The Efficiency of Clusters on Networks and Their Robustness

**DOI:** 10.3390/e28080865

**Published:** 2026-08-01

**Authors:** Silu Wang, Qingyuan Hu, Jiao Gu

**Affiliations:** School of Mathematics and Data Science, Jiangnan University, Wuxi 214122, Chinaqingyuanhu@jiangnan.edu.cn (Q.H.)

**Keywords:** cluster efficiency, complex networks, attack strategies, robustness, topology optimization

## Abstract

Cluster structures are widespread in complex networks, yet conventional network-level measures do not distinguish the accessibility provided inside a cluster from that provided through its external links. This study asks how these two topological contributions can be measured consistently and how rapidly they deteriorate under different node-removal mechanisms. We define internal and external cluster efficiency by combining edge volume, relative cluster size, and a harmonic shortest-path factor based on the mean reciprocal distance rather than the reciprocal of an arithmetic mean distance. The measures are evaluated together with a Cluster Robustness Index (CRI) and Structural Resilience Entropy (SRE) on Barabási–Albert and Lancichinetti–Fortunato–Radicchi networks and five empirical network topologies under six attack strategies. The results show that smaller clusters are generally more vulnerable to attacks on central nodes, whereas larger and less centralized clusters retain more topological efficiency. A source-level audit also shows that replacing the reciprocal of the arithmetic mean distance by the arithmetic mean of reciprocal distances changes most normalized trends only slightly; the clearest quantitative changes occur in the weighted-average efficiency panels. Intra-cluster edge addition increases internal efficiency by 1–5% and CRI-based robustness by up to 11.9%, while cross-cluster edge rewiring improves external efficiency by up to 2.45% and CRI-based robustness by up to 2.33% under the tested targeted attacks. The reported quantities are structural proxies derived from unweighted network topology; they do not represent observed flow, transmission speed, recovery dynamics, or domain-specific functionality. The framework therefore supports cluster-level vulnerability diagnosis and topology-oriented reinforcement without making claims beyond the available network data.

## 1. Introduction

Complex networks provide structural representations of social, biological, economic, and infrastructure systems [1,2,3,4]. A recurrent feature is a clustered or modular organization, in which connections are denser within groups than between groups [5,6]. Such organization can improve local cohesion while concentrating inter-cluster dependence on a limited set of bridges. Recent work further shows that strong modular organization can create a broad vulnerability to targeted attacks across networks with very different degree distributions [7]. This tension motivates a cluster-level analysis that separates internal accessibility from external accessibility.

Network efficiency is commonly defined through reciprocal shortest-path distances [8]. The harmonic construction limits the influence of very long paths and remains meaningful when some node pairs are disconnected. Global efficiency and average shortest-path measures, however, aggregate the entire graph and do not identify which cluster contributes to an observed loss. Existing community-vulnerability measures use centrality, entropy, curvature, or modularity to diagnose structural weakness [9,10,11,12], but they do not provide a unified pair of internal and external cluster-efficiency measures.

Robustness enhancement has been studied through degree-preserving rewiring, optimization-based reconfiguration, load redistribution, and community-preserving edge modification [13,14,15,16]. Link-addition studies show that a limited number of carefully selected edges can improve connectivity and robustness in single and interdependent networks [17,18,19,20]. Rewiring and loop-oriented designs can also mitigate targeted attacks and promote onion-like robust structures [21,22,23,24]. These studies motivate our two interventions, but our objective is narrower: to improve cluster-level internal or external efficiency under a fixed topological budget rather than to claim a universally optimal network design.

The central research question is: Can cluster-level internal and external accessibility be measured with a harmonic-distance formulation and used to diagnose which structural component fails under random and targeted node removal? We use the following terminology throughout. Efficiency denotes a shortest-path-based topological quantity; vulnerability denotes the relative loss after a specified removal; robustness denotes retention of structure or efficiency over an attack trajectory; and SRE describes the distribution of component sizes after fragmentation. Because no repair or temporal recovery process is modeled, “resilience” is not used as a synonym for recovery.

The main contributions are threefold. First, we define internal and external cluster efficiencies that combine cluster scale, edge volume, and the mean reciprocal distance within the relevant reachable node-pair set. Second, we integrate these measures with CRI, SRE, and area-under-the-curve (AUC) summaries to distinguish structural fragmentation from loss of internal or external accessibility. Third, we test two budgeted topology modifications—intra-cluster edge addition and cross-cluster edge rewiring—on synthetic and empirical network topologies. The empirical examples are interpreted only as topological case studies; labels such as trade, collaboration, or transportation identify the source network and do not convert the structural metric into a measured economic or operational performance variable.

Using six node-removal strategies, we evaluate 50 Barabási–Albert (BA) networks, 50 Lancichinetti–Fortunato–Radicchi (LFR) benchmark networks, and five empirical networks. The remainder of the paper is organized as follows. Section 2 defines the metrics, attack protocols, and optimization strategies. Section 3 reports the synthetic and empirical results. Section 4 summarizes the conclusions and limitations.

## 2. Methods

To simulate scale-free networks, we use the Barabási–Albert (BA) model, which captures the preferential attachment mechanism common in social, biological, and transportation networks. For statistical robustness, we generate 50 independent BA realizations, each with 1000 nodes and an average degree of 8. These networks exhibit power-law degree distributions, confirming their scale-free topology.

Scale-free networks often have intricate community organizations, making cluster detection essential. We apply the Louvain algorithm [25], which effectively uncovers hierarchical cluster structures. The algorithm operates in two iterative stages driven by modularity optimization: first, it maximizes modularity by locally reassigning nodes to neighboring clusters; second, it aggregates clusters into super-nodes to form a coarse-grained network. This cycle repeats until modularity converges. Using modularity as the objective function ensures rapid convergence and stable partitions, yielding high clustering efficacy for scale-free networks.

### 2.1. Network Efficiency

For an unweighted graph G=(V,E) with N=|V|, the Latora–Marchiori global efficiency is

(1) $$E_{\mathrm{glob}}=\frac{1}{N(N-1)}\sum_{i\neq j\in V}\frac{1}{d_{ij}},$$

where $d_{ij}$ is the shortest-path length and $1/d_{ij}=0$ when *i* and *j* are disconnected. This arithmetic mean of reciprocal distances is a harmonic-type accessibility measure and should not be confused with the reciprocal of the arithmetic mean distance.

### 2.2. Mean Topological Distance

For comparison with earlier work [26], we also report the mean finite shortest-path length

(2) $$L=\frac{1}{\left|P\right|}\sum_{(i,j)\in P}d_{ij},$$

where $P=\{\left(i,j\right):i<j,\;d_{ij}<\infty \}$. *L* is a distance statistic, not an efficiency: smaller *L* indicates greater topological compactness. It is included only as a comparator and is labeled separately from $E_{\mathrm{glob}}$ in the revised figures.

### 2.3. Internal Efficiency of a Cluster

Let $C_{k}\subseteq V$ be cluster *k*, $n_{k}=\left|C_{k}\right|$, $e_{k}$ the number of edges with both endpoints in $C_{k}$, and $P_{k}^{\mathrm{in}}=\left\{\left(i,j\right):i<j,\;i,j\in C_{k},\;d_{ij}^{\left(k\right)}<\infty \right\}$ the set of reachable unordered node pairs in the induced subgraph $G[C_{k}]$. We define

(3) $$H_{k}^{\mathrm{in}}=\frac{1}{|P_{k}^{\mathrm{in}}|}\sum_{\left(i,j\right)\in P_{k}^{\mathrm{in}}}\frac{1}{d_{ij}^{\left(k\right)}},\;I_{k}=e_{k}H_{k}^{\mathrm{in}}\frac{n_{k}}{N}.$$

If $P_{k}^{\mathrm{in}}$ is empty, $H_{k}^{\mathrm{in}}=0$. The factor $H_{k}^{\mathrm{in}}$ measures topological compactness through the *mean reciprocal distance*; $e_{k}$ represents internal edge volume, and $n_{k}/N$ weights the cluster by its share of the original network. This definition contains no transmission-rate or temporal variable, so $I_{k}$ is interpreted as structural internal efficiency rather than transmission speed, collaboration quality, or realized flow.

For plots that compare networks or clusters with different raw scales, $I_{k}$ is min–max normalized within the stated experiment. Raw values are retained for attack ratios and AUC calculations so that normalization does not change the ordering along a trajectory.

### 2.4. External Efficiency of a Cluster

Let $m_{k}$ be the number of edges with exactly one endpoint in $C_{k}$, and let $P_{k}^{\mathrm{out}}=\left\{\left(i,j\right):i\in C_{k},\;j\in V\setminus C_{k},\;d_{ij}<\infty \right\}$. The external harmonic accessibility and external cluster efficiency are

(4) $$H_{k}^{\mathrm{out}}=\frac{1}{|P_{k}^{\mathrm{out}}|}\sum_{\left(i,j\right)\in P_{k}^{\mathrm{out}}}\frac{1}{d_{ij}},\;E_{k}=m_{k}H_{k}^{\mathrm{out}}\frac{n_{k}}{N}.$$

If no reachable cross-cluster pair exists, $H_{k}^{\mathrm{out}}=0$. Thus, $m_{k}$ represents the external edge volume, $H_{k}^{\mathrm{out}}$ represents cross-cluster topological accessibility, and $n_{k}/N$ is the same original-size weight used in $I_{k}$. A high $E_{k}$ therefore means that the cluster has many and relatively short topological routes to the remainder of the graph; it does not by itself establish stronger cooperation, higher trade, or better operational performance.

The reachable-pair convention in Equations (3) and (4) matches the supplied simulation workflow. Fragmentation is not ignored: loss of nodes, edges, or reachable pairs reduces $e_{k}$, $m_{k}$, and often the harmonic factors, while complete disconnection sets the corresponding factor to zero. GCC, CRI, and SRE separately record graph fragmentation. The accompanying Python 3.14.6 package also implements a Latora-style “zero for unreachable pairs” option as a sensitivity check and includes hand-verifiable tests for both conventions.

### 2.5. Robustness Assessment Approach

Our analysis evaluates progressive node-removal ratios from the intact graph to complete removal. Two classes are considered: random failures and centrality- or walk-based targeted removals. For degree, betweenness, k-shell, and bridge attacks, nodes are ranked on the intact graph and the top-ranked fraction is removed at each ratio; the degree-biased random walk is generated sequentially. This statement matches the released Python implementation and avoids implying adaptive re-ranking when it is not performed.

**Random failures**: At each step, a node is uniformly chosen and removed with all its edges, independent of any attributes. Results are averaged over 300 independent repetitions.

**Targeted attacks**: Nodes are removed according to the baseline ranking induced by the specified criterion; attack curves are evaluated at the same removal ratios for all strategies.

Targeted strategies are divided into two categories: global-information-based and local-information-based. Global strategies include degree centrality [27], betweenness centrality, bridging centrality [28], and k-shell decomposition. The local strategy is a degree-biased random walk [29]. These strategies are abstract topological stress tests. No specific accident, policy intervention, epidemic, or cascading-flow mechanism is simulated.

- *Degree centrality*: Removes nodes with highest degree, targeting the most connected cores. Effective for disrupting global connectivity but may miss systemic impacts beyond immediate neighbors.
- *Betweenness centrality*: Measures path-based centrality. Nodes with high betweenness lie on many shortest paths. It is defined as
  (5) $$BC\left(i\right)=\sum_{s\neq i\neq t}\frac{g_{st}^{i}}{g_{st}}$$
  where $g_{st}^{i}$ is the number of shortest paths from node *s* to node *t* via node *i*, and $g_{st}$ represents the total number of shortest paths from node *s* to node *t*.
- *Bridging centrality*: Targets “bridge nodes” that are critical for inter-cluster connectivity. Their removal increases average path length or splits the network. The bridging centrality of node *i*, denoted BRC(i), is defined as follows:
  (6) $$\mathrm{BRC}\left(i\right)=\frac{\tau (i)}{\sum_{\begin{array}{c} j\neq i\in G \end{array}}\tau \left(j\right)}$$
  Here, τ(i) represents the bridging value of node *i*, defined as $\tau \left(i\right)=\frac{m_{i}^{\mathrm{inter}}}{k_{i}}$, where $k_{i}$ is the total degree of node *i* and $m_{i}^{\mathrm{inter}}$ denotes the number of edges connecting node *i* to nodes in other clusters. A high ratio indicates a dominant role in cross-cluster communication.
  This strategy directly tests external efficiency robustness: removing bridge nodes reveals clusters that rely heavily on few external links. Results can guide reinforcement, e.g., redistributing connections or creating alternative bridges.
- *K-shell*: Assigns each node a k-shell index based on core–periphery position. Attacks start from the innermost core (highest k-shell). Removal of core nodes severely disrupts global structure but may overlook locally influential nodes.
- *Degree-biased random walk*: Uses only local information. Starting from a random node, the next attack target is chosen among its neighbors with probability proportional to their degree. The walk continues until no neighbors remain, then restarts from a random surviving node. This mainly disrupts local connectivity with limited global impact. Results are averaged over 300 independent repetitions.

### 2.6. The Vulnerability of Cluster

We quantify node importance through cluster vulnerability, defined as the relative efficiency loss when removing critical nodes:

(7) $$V_{internal/external}=\frac{P-T}{P}$$

where $V_{internal}$ denotes the vulnerability of a cluster’s internal efficiency, and $V_{external}$ denotes the vulnerability of its external efficiency. Here, *P* represents the cluster’s internal (or external) efficiency before node removal, and *T* is the corresponding efficiency after the removal of nodes from that cluster.

### 2.7. Indicators for Cluster Structure and Robustness

To assess cluster robustness under systematic node removal, we combine connectivity, modularity, partition stability, path-based efficiency retention, and fragmentation. These quantities are reported separately or through the comprehensive cluster robustness index; no repair process or domain-level function is modeled.

#### 2.7.1. Structural Integrity

The structural integrity of the network at the cluster level after an attack (e.g., node removal) is reflected in three fundamental aspects:

1. Network Connectivity
  The connectivity of a network is measured in terms of the relative size of the maximally connected subgraph (GCC):
  (8) $$GCC\left(r\right)=\frac{\left|LCC(r)\right|}{N}$$
  where |LCC(r)| is the number of nodes in the giant component after removing a fraction *r* of nodes, and *N* is the initial total number of nodes in the network. A slower decline in GCC(r) indicates better macro-scale integrity.
2. Modularity
  The relative change in modularity (*Q*) reflects preservation of cluster cohesion:
  (9) $$Q_{\mathrm{ratio}}\left(r\right)=\frac{Q(r)}{Q(0)}$$
  where Q(r) is the modularity after attack. The ratio quantifies how well intra-cluster edge density advantage over inter-cluster links is maintained.
3. Cluster Structure Stability
  Normalized Mutual Information (NMI) is used to measure the similarity between the community segmentation result $C_{r}$ and the initial segmentation $C_{0}$ of the network after the attack.
  (10) $$\mathrm{NMI}\left(C_{0},C_{r}\right)=\frac{2I(C_{0},C_{r})}{H\left(C_{0}\right)+H\left(C_{r}\right)}$$
  where *I* is the mutual information and *H* is the entropy. Higher NMI implies more stable cluster membership.

Based on the above three basic metrics, we construct a comprehensive cluster robustness index (CRI) for any attack ratio *r*:

(11) $$CRI\left(r\right)={\left(\frac{GCC(r)}{GCC(0)}\right)}^{m}\cdot {\left(\frac{Q(r)}{Q(0)}\right)}^{q}\cdot {\left(\mathrm{NMI}(C_{0},C_{r})\right)}^{l}$$

with m+q+l=1. Here, m=q=l=1/3. The area under the curve (AUC) summarizes retention over the full attack trajectory:

(12) $$\mathrm{AUC}-\mathrm{CRI}=\int_{0}^{1}CRI\left(r\right)dr$$

A larger AUC−CRI value indicates that the network’s cluster structure maintains its comprehensive integrity better in the face of progressive attacks.

#### 2.7.2. Structural Resilience Entropy

Before introducing Structural Resilience Entropy (SRE), we clarify its relation to classical percolation theory. Percolation theory studies the emergence of a giant connected component under node or edge removal [30], and is commonly used to determine whether a network remains globally connected after damage. Recent extensions to multiplex networks and spatial networks have uncovered complex phase behaviors, including discontinuous transitions and reentrant phases [31,32]. However, percolation theory is inherently binary, it only indicates the presence or absence of a giant component. This binary nature fails to capture the internal structure of fragmentation. Two networks lacking a giant component may differ radically: one may retain several medium-sized connected components, while the other shatters into numerous isolated nodes. These scenarios imply different residual fragmentation patterns, which the largest-component indicator alone cannot distinguish.

This limitation motivates SRE. Relying solely on the largest component overlooks critical fragmentation details. SRE quantifies the uniformity of fragment sizes across all connected components, measuring structural disorder after an attack [33]. Unlike traditional metrics that treat all sub-threshold states as failures, SRE distinguishes a polarized structure (dominated by one component) from a highly fragmented state (many components of similar size). Formally, for a given attack ratio *r*, let the remaining network consist of *M* connected components with sizes $s_{i}\;\left(i=1,\ldots ,M\right)$. The normalized size distribution is $p_{i}=s_{i}/\sum_{j=1}^{M}s_{j}$. Then,

(13) $$SRE\left(r\right)=-\frac{1}{lnM}\sum_{i=1}^{M}p_{i}\log p_{i}.$$

#### 2.7.3. Path-Based Efficiency Retention

Structural integrity does not equal path-based efficiency. We therefore track the retention of internal efficiency $I_{k}$ and external efficiency $E_{k}$ under attack.

For a network with multiple clusters, let I(r) and E(r) denote the size-weighted averages defined below. Their normalized AUC values quantify retention of internal and external path-based efficiency:

(14) $$\mathrm{AUC}-I=\int_{0}^{1}\frac{I(r)}{I(0)}dr,\;\mathrm{AUC}-E=\int_{0}^{1}\frac{E(r)}{E(0)}dr.$$

AUC−I and AUC−E measure the average retention of internal and external topological efficiency during attacks, respectively. These two metrics are analyzed alongside AUC−CRI and SRE to determine whether an attack primarily disrupts structure, impairs efficiency, or both.

Together, AUC–CRI, SRE, AUC–*I*, and AUC–*E* provide complementary, rather than interchangeable, views: structural retention, fragmentation balance, internal-efficiency retention, and external-efficiency retention.

### 2.8. Strategies for Enhancing the Robustness of Network Clusters

We consider two budgeted topology modifications. Their purpose is to test whether the diagnosed internal or external weakness can be reduced with a small number of changes; they are not claimed to be globally optimal.

#### 2.8.1. Intra-Cluster Edge Addition Strategy

Within each cluster, nodes are ranked by internal degree and the bottom 20% form the peripheral set $V_{\mathrm{peripheral}}$. Let $G_{0}=\left(V,E_{0}\right)$ denote the intact network before any robustness intervention. The total edge-addition budget is

(15) $$B_{\mathrm{add}}=\left\lfloor 0.05|E_{0}|+\frac{1}{2}\right\rfloor ,$$

where $|E_{0}|$ is the number of edges in the intact network and the floor expression rounds $0.05|E_{0}|$ to the nearest nonnegative integer. The fraction 0.05 is fixed before observing the outcomes and represents a modest 5% intervention budget: it is large enough to permit measurable structural reinforcement, small enough to avoid redesigning the network, and is applied uniformly across all networks for comparability. It is a controlled experimental setting rather than a theoretically optimal value. The integer budget is distributed among clusters in proportion to their original sizes. At each step, a node $u\in V_{\mathrm{peripheral}}$ is sampled and connected to a non-neighbor *v* in the same cluster with minimum current internal degree, preferring another peripheral node when possible. Degrees are updated after each addition.

The low-degree rule is motivated by minimum-degree link-addition studies, which find that reinforcing low-degree nodes can reduce degree concentration and improve robustness [17]. Our method is similar in selecting low-degree endpoints, but differs in three respects: additions are restricted to the same detected cluster, the budget is allocated by cluster size, and the second endpoint is chosen by current internal degree rather than by a global longest-distance rule. Selecting peripheral nodes therefore directly targets the internal redundancy deficit identified by $I_{k}$ while limiting new hub formation.

#### 2.8.2. Cross-Cluster Edge Rewiring Strategy

For each cluster $C_{k}$, we identify the external cluster receiving the largest number of its cross-cluster edges. Those dominant-dependency edges form the candidate set. A budget equal to 10% of that candidate set is rewired: an edge (u,v) to the dominant external cluster is removed and $\left(u,v^{\prime }\right)$ is added to a node in a different external cluster, provided the new edge does not already exist. The number of edges is preserved.

This strategy follows the general robustness rationale of loop enhancement, bypass rewiring, and onion-like diversification [21,22,23,24], but its objective is specifically to reduce concentration of a cluster’s external dependencies. It does not preserve every node degree and should therefore be interpreted as a constrained heuristic rather than a degree-preserving optimum.

## 3. Results and Discussion

### 3.1. Comparison of Efficiency Indicators

**Figure 1:**
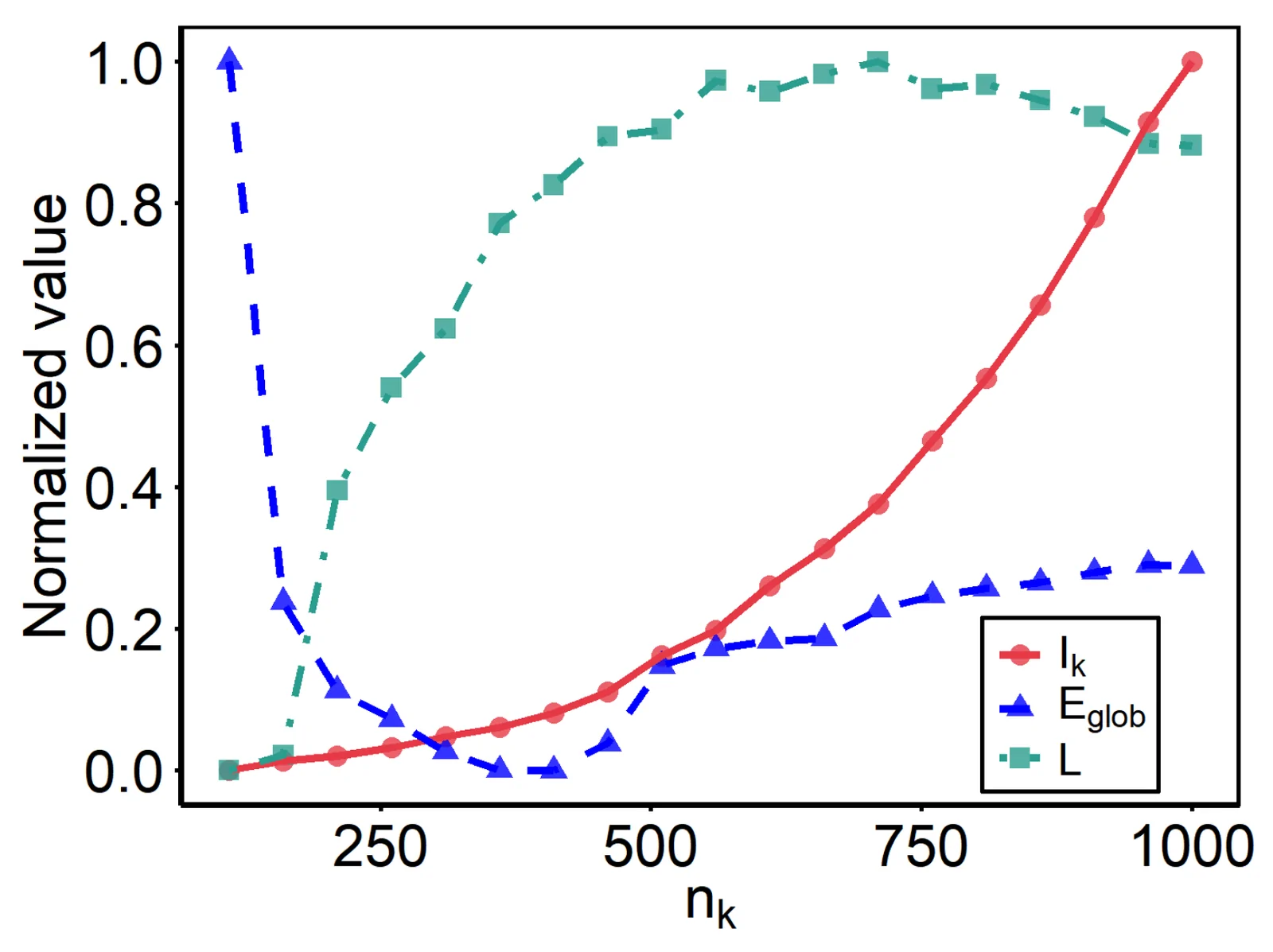
Normalized comparison for one BA growth experiment (N=1000, attachment parameter m=4). The red curve is $I_{k}$ for the growing cluster, the blue curve is global efficiency $E_{\mathrm{glob}}$, and the green curve is the finite-pair mean distance comparator *L*; each curve is min–max normalized separately, so only within-curve trends should be compared.

Figure 1 compares $I_{k}$, global efficiency $E_{\mathrm{glob}}$, and the finite-pair mean distance *L* as the tracked BA cluster grows. Because each series is min–max normalized separately, only the direction and shape of each series should be interpreted; their vertical magnitudes are not directly comparable. In this experiment, $E_{\mathrm{glob}}$ decreases after the early growth stage, whereas *L* generally increases, which is consistent with longer shortest paths in a larger graph.

The increase in $I_{k}$ is a consequence of its explicit edge-volume and relative-size factors together with the harmonic-distance factor; it is not evidence that a larger institution or economic bloc necessarily performs better. Figure 1 therefore illustrates how the proposed topological quantity responds to scale in one controlled growth setting, rather than establishing a domain-specific law.

The comparison also clarifies the intended role of $I_{k}$: it complements a path-only statistic by accounting for the amount of internal connectivity and the cluster’s share of the original network. Any operational interpretation would require additional flow, capacity, cost, or temporal data.

### 3.2. Relationship Between Cluster Efficiency and Structural Properties

Based on the definitions of internal and external cluster efficiency, we examine their relationships with cluster size and connection density. These relationships deepen theoretical understanding and offer practical insights for network design and robustness enhancement.

**Figure 2:**
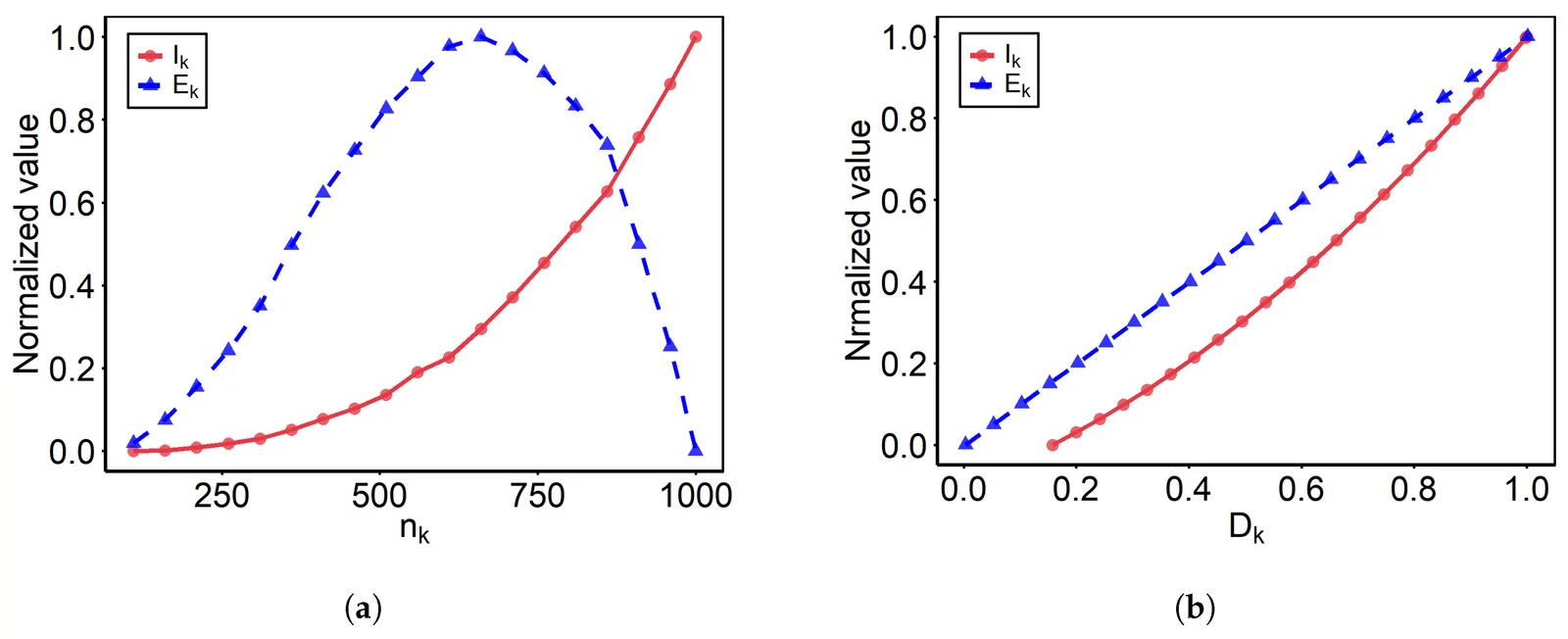
Cluster-level sensitivity in one BA growth experiment (N=1000, m=4). (**a**) Separately normalized $I_{k}$ and $E_{k}$ versus the tracked cluster size $n_{k}$. (**b**) Separately normalized $I_{k}$ and $E_{k}$ versus internal edge density $D_{k}=2e_{k}/\left[n_{k}\left(n_{k}-1\right)\right]$. The curves describe the tracked cluster and are not averages over all clusters.

In the tracked BA growth experiment, normalized $I_{k}$ increases nonlinearly with $n_{k}$ and approximately monotonically with $D_{k}$ over the sampled range (Figure 2). These empirical curve shapes are descriptive and are not presented as universal scaling laws or optimization guarantees.

Normalized $E_{k}$ is non-monotone with $n_{k}$ in this experiment and increases with $D_{k}$. The first pattern reflects the combined effects of the number of external edges, the reachable cross-cluster distances, and the size weight in Equation (4); the second indicates that denser external connectivity is associated with higher topological external efficiency under the tested construction.

### 3.3. Simulation

To simulate and evaluate the robustness of cluster efficiency, we generated 50 Barabási–Albert (BA) scale-free networks, each with 1000 nodes and an average degree of 8. Community detection was performed using the Louvain algorithm. While these synthetic BA networks effectively capture the hub and spoke topology common in real systems, such as airline networks where a few major hubs connect to numerous smaller airports, they lack explicit modular or geographic clustering. To address this limitation, we also employed the LFR benchmark model, a widely adopted framework for testing community detection algorithms. Specifically, we constructed LFR networks with the same node count and average degree (*N* = 1000, *k* = 8), setting the mixing parameter μ = 0.1 and allowing community sizes to range from 20 to 120. This configuration more closely approximates the modular and heterogeneous nature of empirical networks [34].

**Figure 3:**
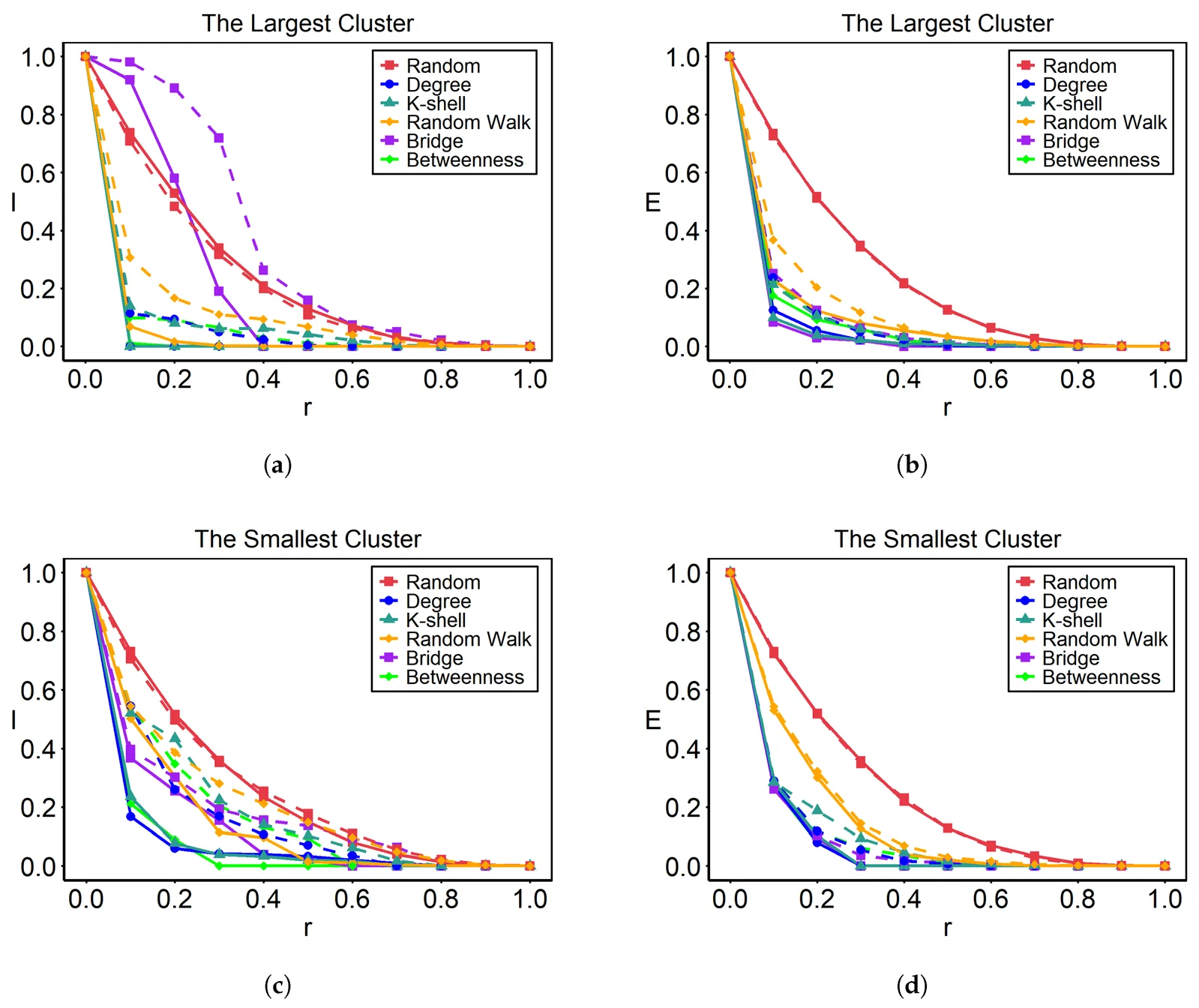
Average changes in robustness for internal and external efficiency of the largest and smallest clusters across 50 scale-free networks and 50 LFR benchmark networks under different attack strategies. Solid lines correspond to LFR benchmark networks, while dashed lines represent scale-free networks.

**Figure 4:**
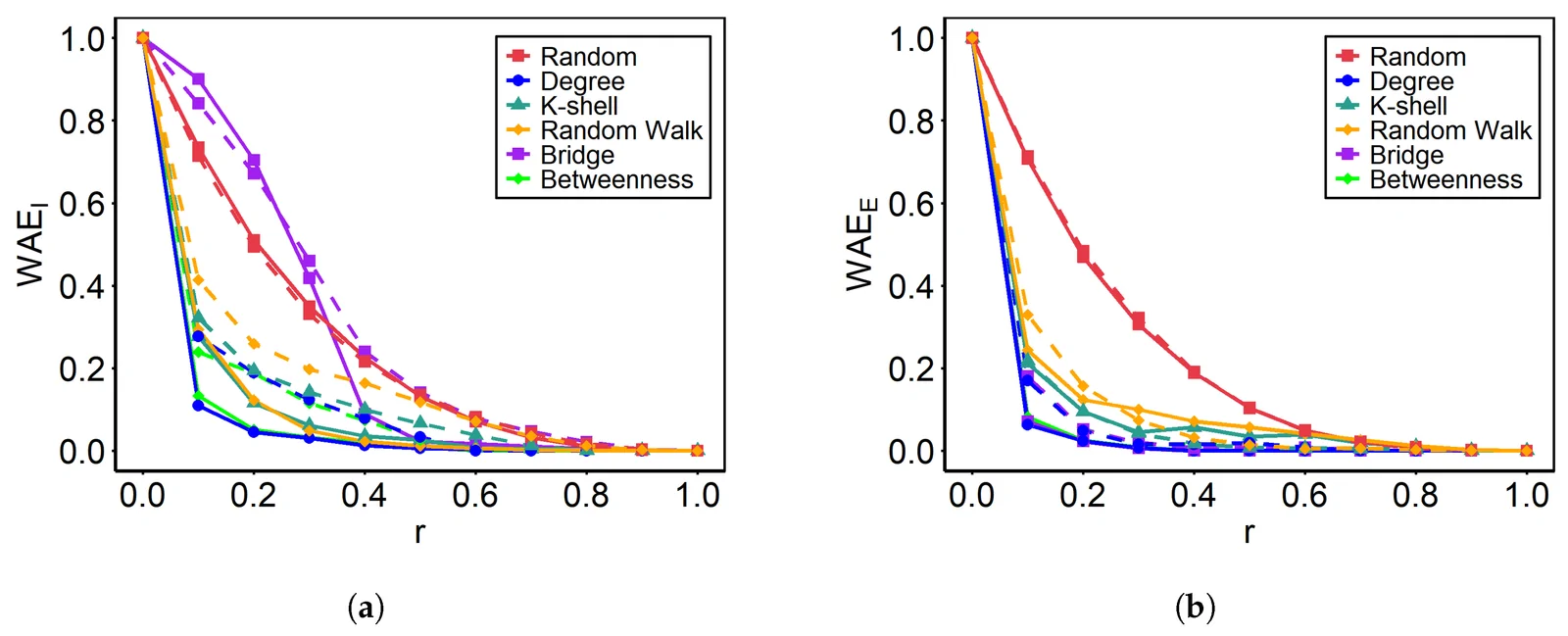
Changes in robustness of the weighted average internal and external efficiency across all clusters in 50 scale-free networks and 50 LFR benchmark networks under different attack strategies. Solid lines correspond to LFR benchmark networks; dashed lines represent scale-free networks.

To bridge the network configurations with our robustness analysis, we subject these models to various targeted and random attack scenarios to evaluate how structural degradation impacts efficiency. For robustness analysis, we first examine two representative clusters from each network type (LFR and BA)—one large and one small, as shown in Figure 3. This allows us to observe how cluster size influences vulnerability under various attack scenarios. Recognizing the limited generalizability of case-specific observations, we extend the analysis to the entire network by comparing two methods for calculating average cluster efficiency. A weighted average calculation method is then selected to assess internal and external cluster efficiency across both network models (Figure 4).

The results in Figure 3 reveal consistent patterns. Random node removal has a negligible impact on cluster efficiency in both BA and LFR networks, regardless of cluster size. In contrast, targeted attacks based on degree, betweenness, or k-shell centrality cause a sharp initial decline in efficiency, affecting both large and small clusters. The degree-biased random walk strategy demonstrates moderate destructive power: its early impact is relatively mild, but it progressively degrades cluster performance by preferentially removing high-degree nodes, placing its overall effect between random failures and more aggressive targeted strategies. Bridge node attack exhibits a distinct pattern: it has the greatest impact on external cluster efficiency, while its impact on internal efficiency is minimal. Since bridge nodes serve as critical connections between clusters, their removal severs inter-cluster links, significantly impairing external efficiency. Most bridge nodes are located near cluster peripheries, which explains their limited influence on internal cluster efficiency.

Comparing the two synthetic network families reveals different metric-retention profiles. In the tested LFR networks, the efficiency of large clusters drops markedly after removing about 10% of nodes under attacks using global centrality information, whereas the tested BA networks retain a nonzero efficiency level after more extensive removal. Degree- and betweenness-based attacks are the most damaging among the evaluated strategies. The lower retention observed for the LFR instances is associated with their stronger modular organization and concentrated within-cluster connectivity; this comparison is specific to the parameter settings used here.

We additionally audited every source-code occurrence of the path factor to distinguish the Latora–Marchiori aggregation $\bar{d^{-1}}={\left|P\right|}^{-1}\sum_{(i,j)\in P}d_{ij}^{-1}$ from the legacy quantity $1/\bar{d}$. The audit confirms the reviewer’s observation that the correction has only a limited visible effect in most panels. First, several computational blocks used $\bar{d^{-1}}$ already, so their numerical outputs did not require substantive revision. Second, CRI and SRE do not contain the path factor. Third, the plotted efficiency-retention curves divide each attacked value by its own intact-network baseline; a common multiplicative shift therefore cancels to a large extent. The most appreciable changes occur in Figure 4, where aggregation over all clusters exposes greater heterogeneity in shortest-path distributions. Thus, the principal consequence of the correction is conceptual and definitional consistency, accompanied by localized quantitative changes rather than a reversal of the attack rankings or the main robustness conclusions.

Although these results provide valuable insights, their scope remains limited to specific clusters. To gain a more general understanding, we will next use a weighted average method to evaluate the average efficiency of clusters across the whole network. Integrating these individual cluster responses into a unified metric is a necessary step for a generalized assessment of the entire system’s structural integrity.

(16) $$WAE_{I}\left(r\right)=\sum_{k=1}^{K}\frac{n_{k}\left(0\right)}{N}R_{k}^{I}\left(r\right),\;WAE_{E}\left(r\right)=\sum_{k=1}^{K}\frac{n_{k}\left(0\right)}{N}R_{k}^{E}\left(r\right),$$

Here, *K* is the number of clusters in the intact network, $n_{k}\left(0\right)$ is the initial size of cluster *k*, $R_{k}^{I}\left(r\right)=I_{k}\left(r\right)/I_{k}\left(0\right)$ is its internal-efficiency retention, and $R_{k}^{E}\left(r\right)=E_{k}\left(r\right)/E_{k}\left(0\right)$ is its external-efficiency retention.

To assess the robustness of clustering efficiency, we employ a scale-weighted average method that assigns greater weight to larger clusters, as these typically have a more significant impact on overall network performance. This weighted approach provides a more representative assessment of robustness at the network level.

A simple average gives every detected cluster the same influence, irrespective of its initial node count. We instead use the fixed initial-size weights in Equation (16) so that the network-level summary represents the efficiency retention of an initially sampled node’s cluster. This is a transparent aggregation convention, not a claim that larger clusters are intrinsically more functional. When cluster sizes are equal, it reduces to the ordinary arithmetic mean.

Further support comes from a comparative analysis of LFR and BA networks (Figure 4). Under all targeted attack strategies, LFR networks experience faster efficiency decline, reflecting higher inherent vulnerability. This stems from their dense intra-cluster and sparse inter-cluster links, which is a pattern often leading to greater fragility. This susceptibility becomes evident when contrasting failure modes: while these networks remain stable under random perturbations, they show a precipitous efficiency decline under targeted attacks. Disruptions focusing on high-degree or high-betweenness nodes induce rapid efficiency loss, highlighting the vital role of topologically central nodes.

**Figure 5:**
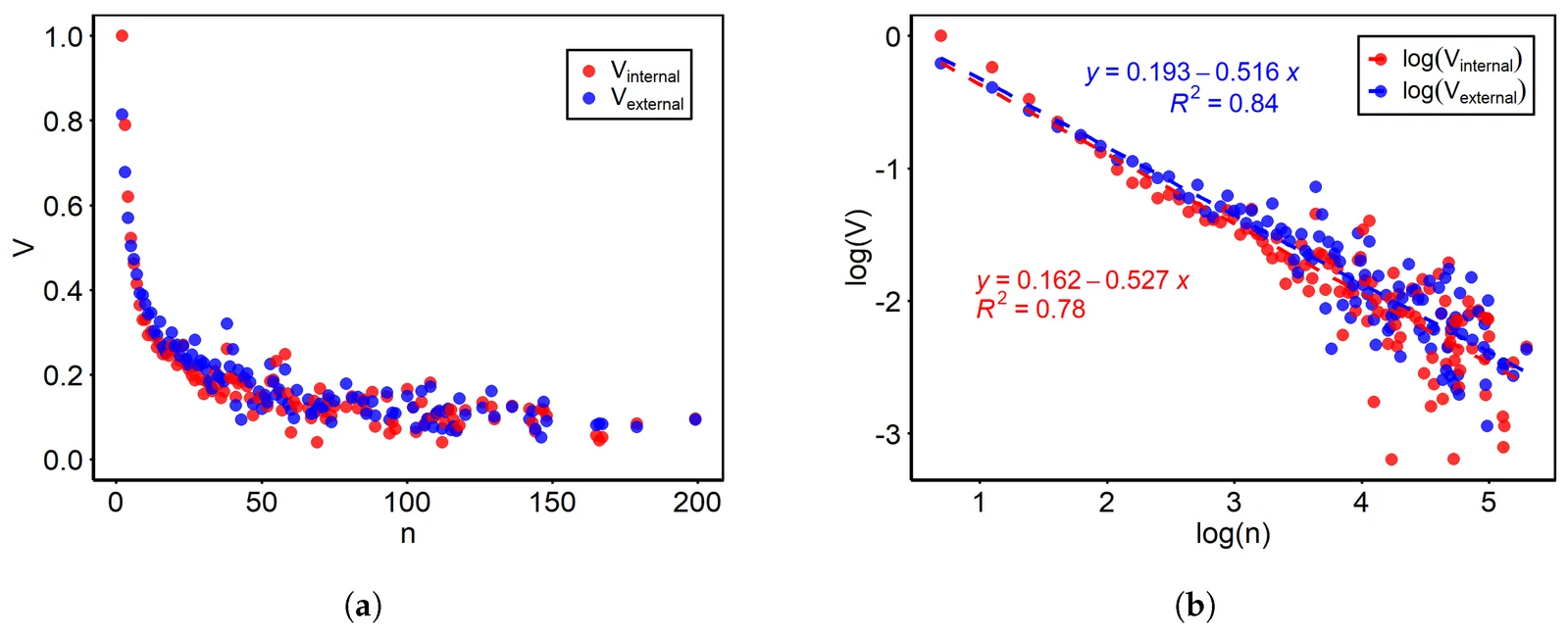
The average vulnerability of the internal and external efficiency of clusters in 50 LFR networks.

To quantify cluster-efficiency vulnerability, we systematically remove the highest-degree node from each cluster across 50 independent LFR networks and compute the average relative efficiency loss. The results, shown in Figure 5, indicate that efficiency vulnerability follows a power-law distribution, with larger clusters displaying lower vulnerability. A log-transformed analysis confirms this inverse relationship, revealing strong linear correlations ($R^{2}=0.78$ for internal efficiency, $R^{2}=0.84$ for external efficiency), suggesting that larger clusters possess greater structural robustness.

For deeper insight, we categorize clusters into large (top 20% in size) and small (bottom 20% in size) groups. As summarized in Table 1, smaller clusters in both LFR and BA networks exhibit substantially higher efficiency losses after targeted node removal, especially in LFR networks. This comparison is attributed to the tested synthetic topologies and parameter settings: smaller modules have fewer alternative paths, while the LFR mixing parameter creates relatively sparse cross-cluster connectivity. No domain-specific collapse mechanism is inferred from these synthetic data.

**Figure 6:**
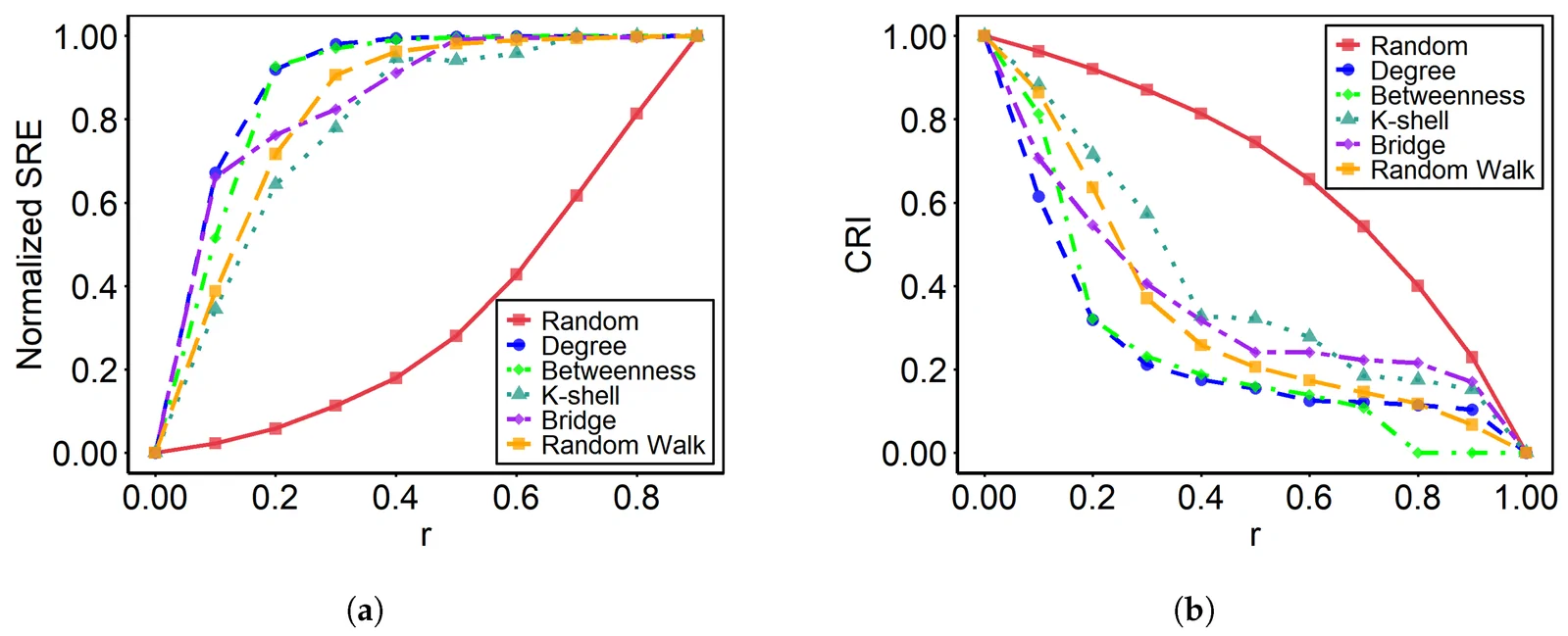
Average variation in Structural Resilience Entropy (SRE) and the Comprehensive Cluster Robustness Index (CRI) across 50 LFR benchmark networks under six attack strategies.

Figure 6 illustrates the evolution of structural resilience entropy (SRE) and the comprehensive cluster robustness index (CRI) in LFR networks under six attack strategies. SRE quantifies patterns of structural fragmentation, whereas CRI evaluates the integrated preservation of connectivity, modularity, and cluster integrity. The SRE curves reveal that different attack strategies induce distinct structural disintegration paths. Under random attacks, SRE rises slowly, indicating a uniform distribution of fragmentation. Under bridge node attacks, SRE increases rapidly in the early stages (r<0.1) and then stabilizes. Conversely, under degree, betweenness, K-shell, and degree-biased random walk attacks, SRE rises rapidly from the outset; at r=0.4, SRE approaches 1, indicating that removing a few critical nodes causes the network to fragment into multiple components of similar size.

The CRI curves summarize the simultaneous loss of connectivity, modularity, and partition stability. Under random attacks, CRI declines gradually and remains near 0.75 at r=0.5, whereas the evaluated targeted attacks produce a concave decline, with degree-based removal yielding the fastest loss. At r=0.2, CRI falls below 0.4 for the most damaging strategies. Together with SRE, these curves show that the tested modular networks are sensitive to removal of internal hubs and cross-cluster bridge nodes; the conclusion is limited to the adopted graph models and attack definitions.

**Figure 7:**
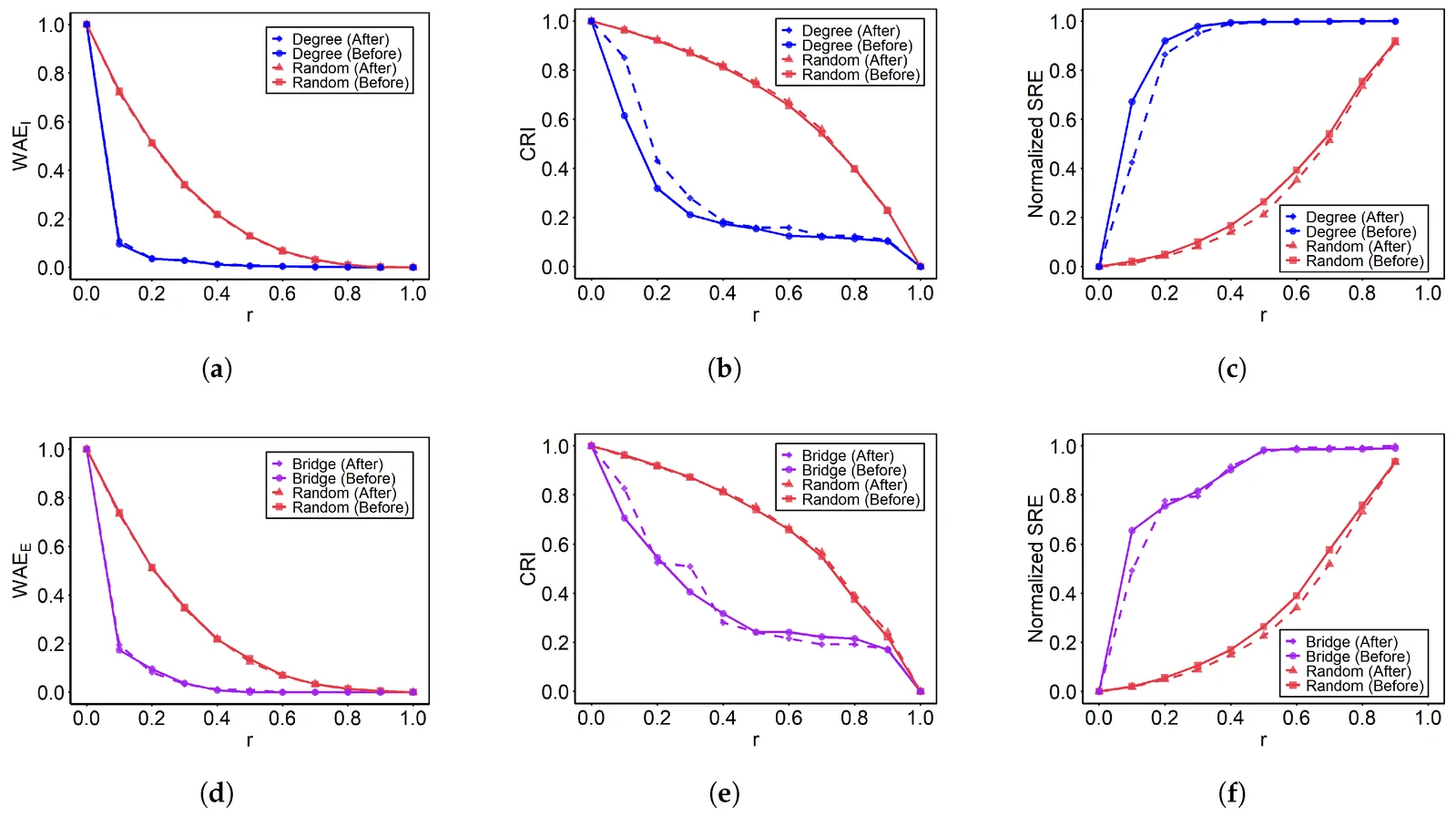
Variation in internal efficiency, external efficiency, SRE, and CRI for an LFR network under two robustness optimization strategies. The first row corresponds to intra-cluster edge addition, and the second row to cross-cluster edge rewiring.

Figure 7 evaluates intra-cluster edge addition and cross-cluster edge rewiring under the budgets defined in Section 2.8. The first strategy shifts the internal-efficiency and CRI curves upward under the displayed random and degree attacks, while the second shifts the external-efficiency curve upward under the displayed bridge attack. The accompanying SRE curves indicate how the component-size distribution changes; they do not model recovery. These results support the narrower conclusion that the two heuristics improve their intended topological targets under the tested settings.

Table 2 provides a quantitative comparison of these strategies. Intra-cluster edge addition increases the area under the internal-efficiency curve (AUC−I) by 2.54% and raises AUC−CRI under degree attack by 19.88%. Cross-cluster edge rewiring increases the area under the external-efficiency curve (AUC−E) by 2.10% and raises AUC−CRI by 2.45% under bridge attack. The blank cells are replaced by dashes because each strategy was evaluated only for its designated efficiency target; the paired AUC–CRI values correspond respectively to degree and bridge attacks.

#### Verificationof Parameter Robustness

To verify the extent to which the proposed Comprehensive Robustness Index (CRI) depends on the choice of weighting parameters and to ensure the generalizability of the evaluation results, we conducted a systematic sensitivity analysis.

**Figure 8:**
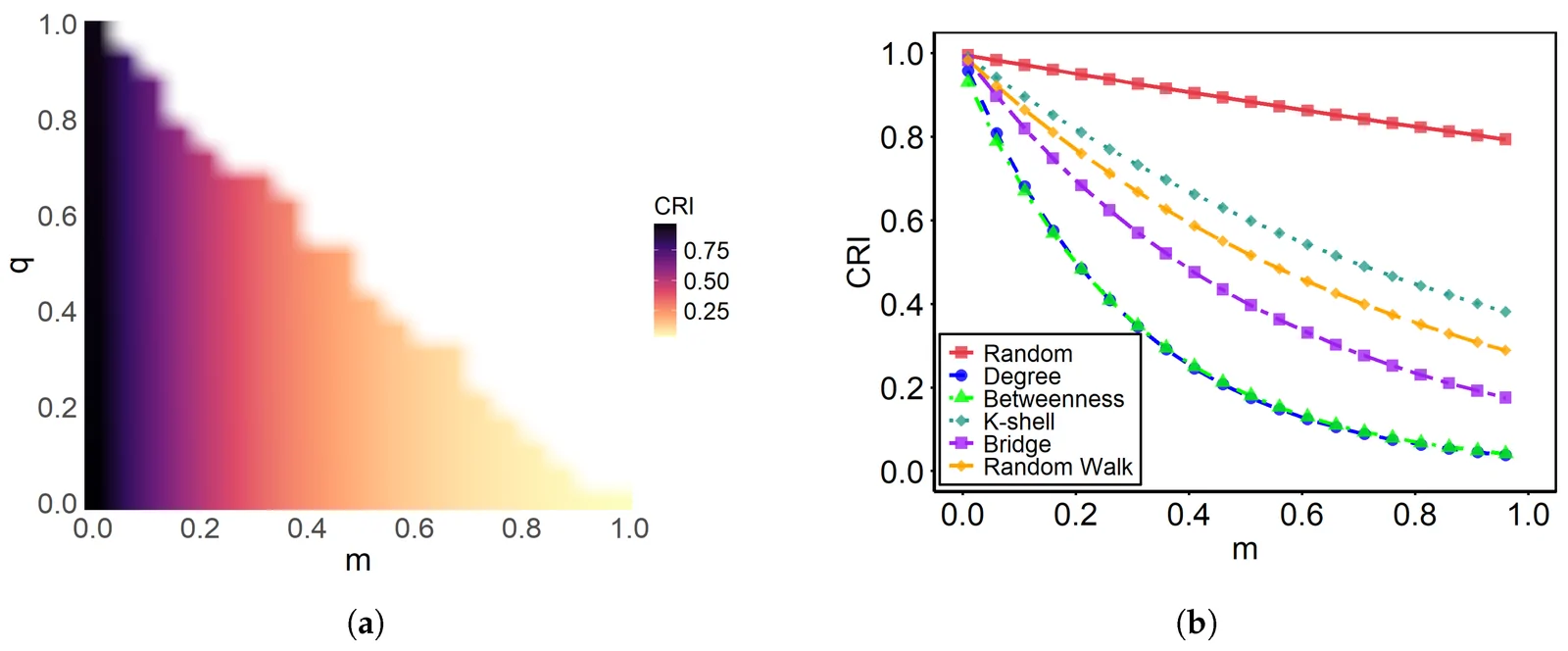
Performance of CRI with different parameter *m*. (**a**) Heatmap of the weight sensitivity analysis for the CRI metric; (**b**) The evolution of CRI values under different attack strategies.

As shown in Figure 8, we performed a grid search of CRI in a two-dimensional weight space, where *m* weights GCC, *q* weights modularity, and l=1−m−q weights NMI. The heatmap shows how CRI varies for a fixed network state. In the displayed range, the value changes less with *q* than with *m* and generally decreases as *m* increases. This occurs because a larger *m* gives greater emphasis to loss of the giant connected component during node removal.

To further quantify this observation, we present the trajectories of CRI values for various attack strategies as m increases when the node removal rate is 20%. The results show that although the CRI values for different strategies fluctuate with changes in weighting, their relative rankings exhibit a high degree of consistency. For example, the curve for the random attack consistently ranks at the top, while the curves for attacks based on betweenness and degree consistently rank at the bottom. This implies that regardless of how weight preferences are adjusted, the core conclusion regarding “which attack strategy causes the greatest damage to the network” remains unchanged.

In summary, the tested weight choices affect the absolute scale of CRI but leave the principal attack ranking unchanged over the evaluated grid. This result supports weight stability for the reported experiment; it does not establish parameter independence outside the tested networks, removal ratio, or weight domain.

### 3.4. Real-World Networks

To rigorously validate our methodology, we conducted benchmark tests on five representative real-world networks organized by their respective domains: social, scientific, biological, transportation, and economic. These include the Polbooks network [35], the Netscience collaboration network [36], the Yeast protein interaction network [37], the China Eastern Airlines (MU) route network, and the World Trade network. Their key topological characteristics are summarized in Table 3 and Table A1.

The structural characteristics of these networks vary significantly across disciplines. In the social domain, the Polbooks network represents political books where edges denote co-purchase relationships among Amazon users; it contains three ground-truth communities (liberal, neutral, and conservative). Representing the scientific domain, the Netscience network models researcher collaborations through co-authorship edges. In the biological domain, the Yeast protein interaction network maps physical interactions between proteins of Saccharomyces cerevisiae. The transportation domain is represented by the MU route network, constructed from 2024 OAG flight data to link operational airports. Finally, in the economic domain, the World Trade network is reconstructed from 2024 UN COMTRADE statistics to encode bilateral trade relationships between sovereign states.

**Figure 9:**
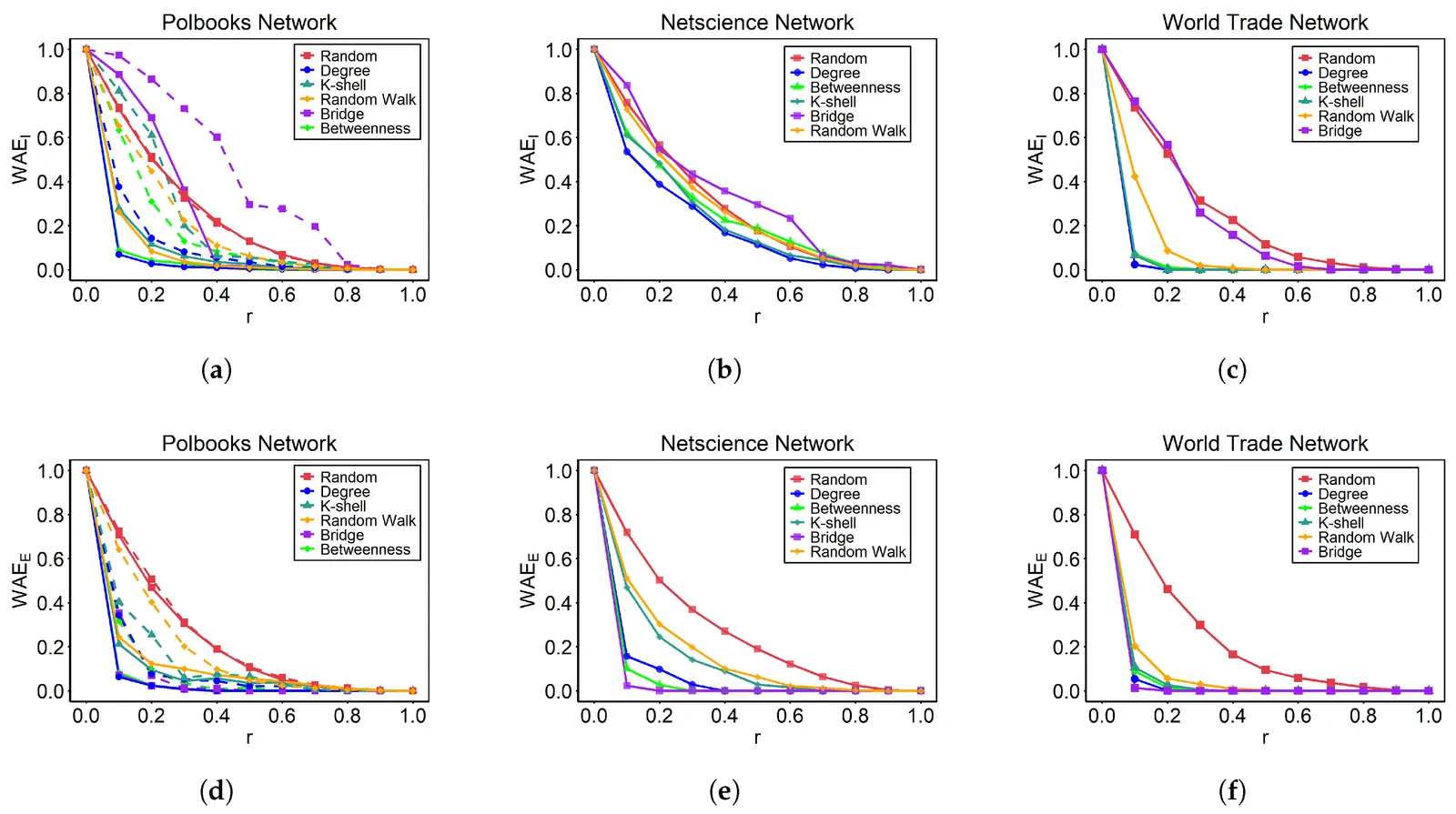
Weighted-average internal and external cluster-efficiency retention in the empirical networks under different attack strategies. The first row shows $WAE_{I}$, and the second row shows $WAE_{E}$.

To maintain methodological consistency, we applied the Louvain algorithm to detect clusters in all empirical networks and evaluated the robustness of cluster-level efficiency under six node-removal strategies. As shown in Figure 9, we compare the weighted average internal and external efficiency across attack scenarios. In the Polbooks network, dashed lines represent GN-detected clusters, while solid lines correspond to the published political-label partition.

Consistent with earlier experiments on LFR and BA networks, the comparative analysis shows that random node failures cause only marginal efficiency loss, whereas targeted attacks lead to a sharp decline. Particularly destructive are degree- and betweenness-based attacks. Degree-biased random walks are more disruptive than random failures but less destructive than global-information attacks. Bridge-node attacks strongly affect external cluster efficiency but have minimal impact on internal efficiency.

For the Polbooks topology, the published political-label partition exhibits higher metric loss under targeted attacks than the algorithmically identified partition. The labeled groups have denser internal connectivity and stronger concentration, which is associated with greater sensitivity to removal of central nodes. This is a structural comparison of two partitions; it does not imply political influence or any other domain-level function.

**Figure 10:**
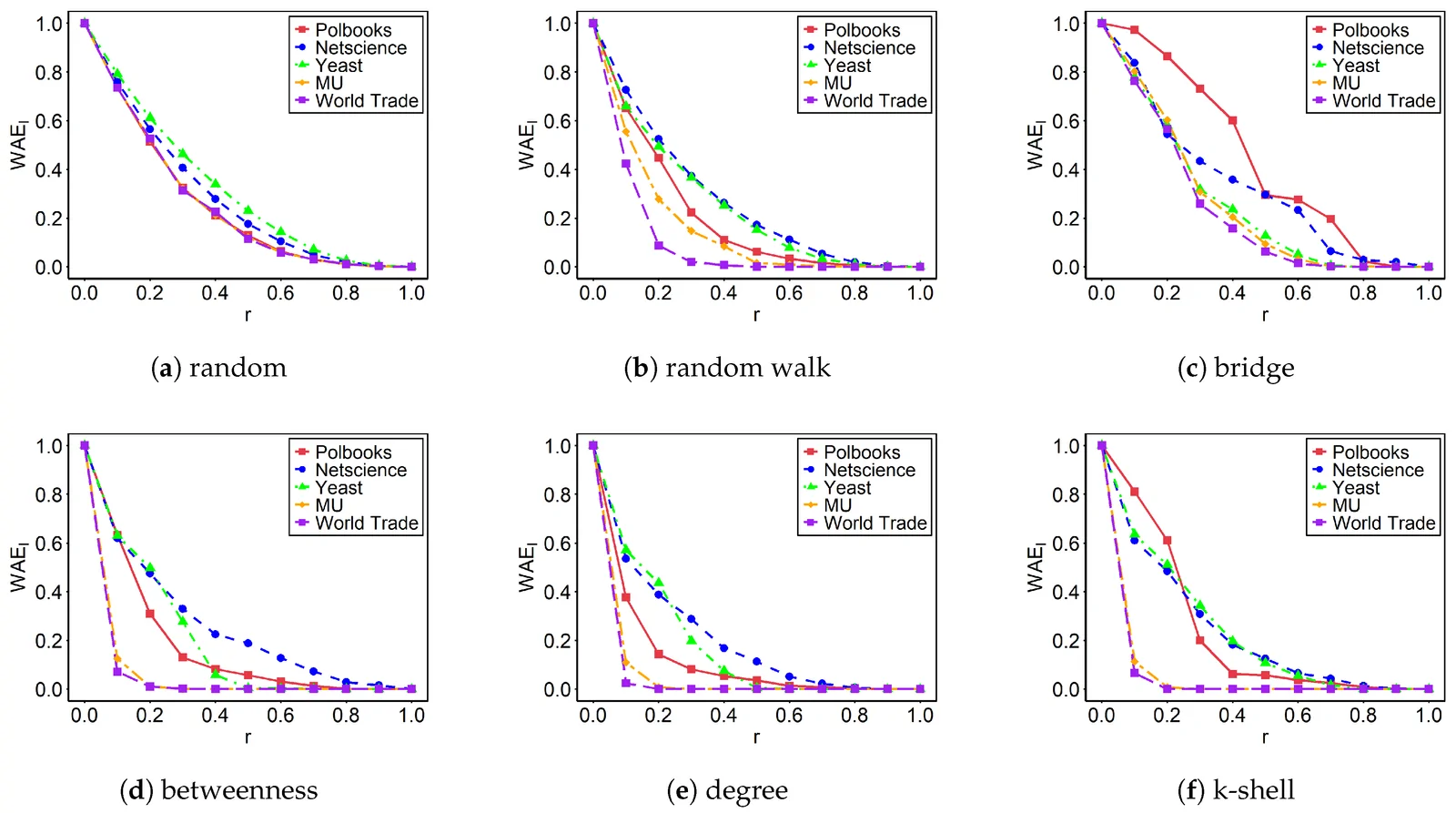
Weighted average internal efficiency $WAE_{I}$ for five empirical topologies under: (**a**) random failure, (**b**) degree-biased random walk, (**c**) bridge attack, (**d**) betweenness attack, (**e**) degree attack, and (**f**) k-shell attack.

Figure 10 shows $WAE_{I}$ for the five empirical topologies. In Table 3, “density” is represented by average degree and edge density; cluster-size balance is summarized by the largest/smallest detected cluster sizes; and internal centralization is assessed jointly from maximum degree and the steepness of targeted-attack curves. These are descriptive associations, not a causal decomposition. No single table entry explains robustness by itself.

Based on their structural characteristics, the five networks can be divided into two groups. The MU and World Trade networks form the first group, characterized by high maximum node degrees and a strong dependence on a small number of core nodes. The remaining three networks—Polbooks, Netscience, and Yeast—constitute the second group. Within this group, the Polbooks network experienced the sharpest decline in internal efficiency due to its relatively small network and cluster sizes. In contrast, the Netscience network exhibited a slower decline across all attack types, confirming that network and cluster size are primary sources of vulnerability.

Cluster size distribution and internal centralization are associated with the differences in robustness observed in these five topologies. Smaller clusters with a few dominant nodes show steep losses under targeted removal. The MU topology retains more of the structural metric than the World Trade topology in the displayed attacks. This difference is consistent with its more balanced detected cluster sizes, but the comparison is descriptive: Table 3 and the attack curves do not establish that cluster-size balance alone causes the observed ranking. Both networks also contain highly central nodes, which helps explain their sensitivity to centrality-based attacks.

Across the five empirical topologies, higher average degree, more balanced detected cluster sizes, and lower internal centralization are associated with greater retention of the proposed metrics. These observations are descriptive associations under the evaluated node-removal procedures, not universal causal rules. They motivate testing whether distributing internal connectivity and avoiding excessive dependence on a few bridge nodes can improve topological retention under a fixed intervention budget.

**Figure 11:**
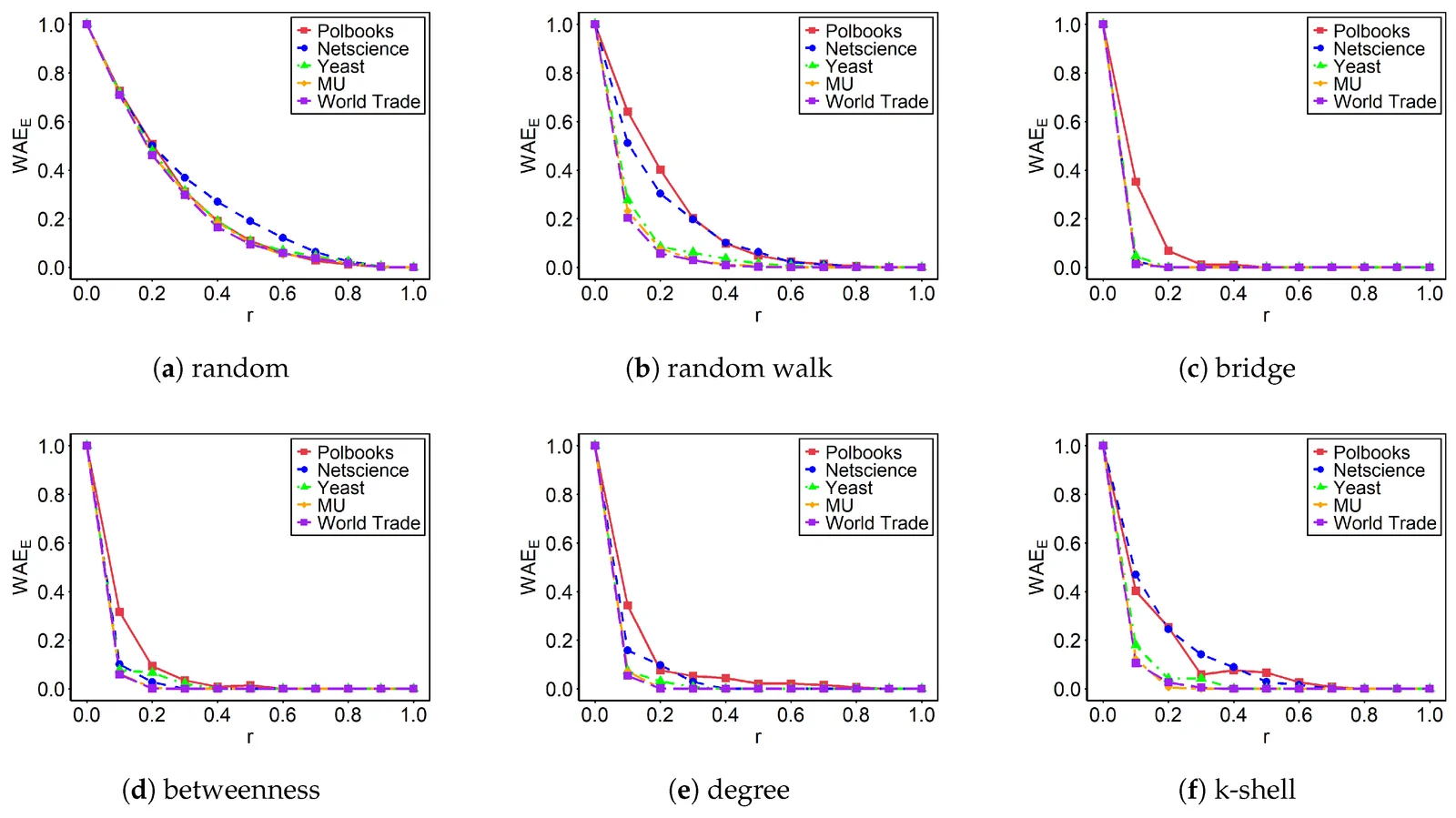
Weighted average external efficiency $WAE_{E}$ for five empirical topologies under: (**a**) random failure, (**b**) degree-biased random walk, (**c**) bridge attack, (**d**) betweenness attack, (**e**) degree attack, and (**f**) k-shell attack.

Figure 11 presents a systematic analysis of external efficiency ($WAE_{external}$) across the five empirical networks. Our findings indicate that the robustness of external cluster efficiency is primarily governed by the global average degree and the distribution pattern of inter-cluster connections. Unlike internal efficiency, external vulnerability depends critically on the concentration and redundancy of bridging links. Networks with high overall connectivity but highly concentrated bridge distributions can exhibit extreme fragility when subjected to targeted attacks.

Average degree and the concentration of inter-cluster links jointly describe the observed differences. Among the lower-degree networks, Polbooks retains the highest external efficiency, whereas the sparse Yeast topology shows the weakest retention. Under bridge-node and betweenness attacks, the World Trade topology declines most rapidly, indicating that many of its inter-cluster shortest paths depend on a limited set of bridge nodes and links. This statement concerns only the topology represented by the supplied graph and does not imply an economic mechanism.

The remaining networks show a more gradual decline, consistent with more balanced detected cluster sizes or more dispersed external links. Thus, both the number and distribution of inter-cluster connections should be considered when evaluating vulnerability to bridge-node removal; the present experiments do not establish a universal design rule.

**Figure 12:**
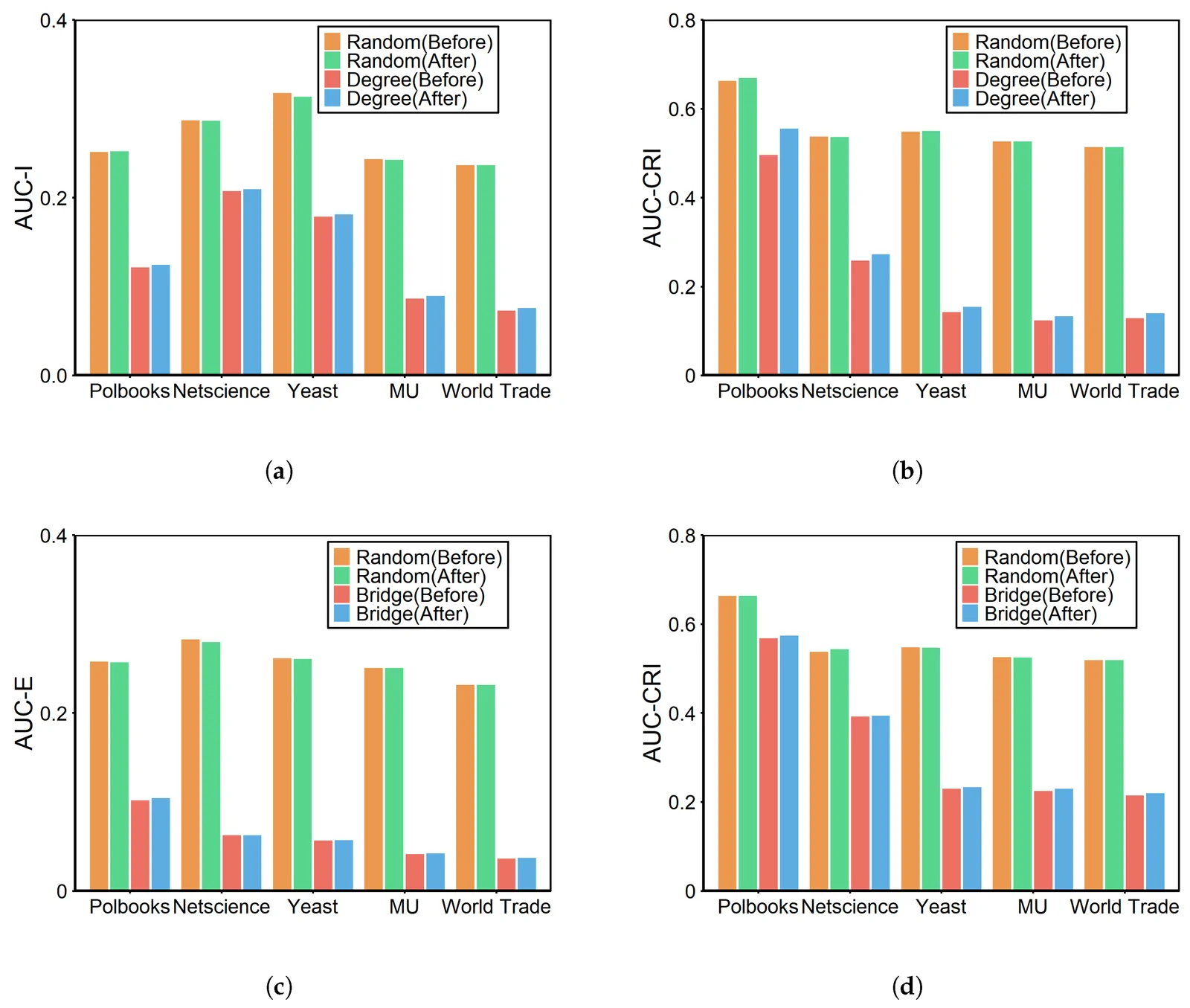
Variation in AUC−I, AUC−E, AUC−CRI across five empirical networks under two robustness optimization strategies. The first corresponds to the intra-cluster edge addition strategy, and the second to the cross-cluster edge rewiring strategy.

Figure 12 and Table 4 provide a quantitative evaluation of the generalizability of the two optimization strategies. The results show that both strategies significantly improve robustness metrics across different network types. Specifically, the intra-cluster edge addition strategy substantially enhances the area under the internal efficiency curve (AUC−I) and the comprehensive cluster robustness index (AUC−CRI) under degree attacks. As shown in Table 4, the improvement in AUC−I ranges from 1% to 5%, with the greatest increase (4.13%) observed for the World Trade network. This indicates that the strategy effectively compensates for the redundancy deficit in internally concentrated structures. Meanwhile, the improvement in AUC−CRI ranges from 5% to 12%, suggesting that enhancing internal connectivity strengthens structural integrity against coordinated attacks.

In contrast, the cross-cluster edge rewiring strategy primarily improves AUC−E and performance under bridge-node attacks. Data in Table 4 indicate that AUC−E improved slightly across all networks, confirming that decentralizing concentrated external connections mitigates strategic vulnerability. While the AUC−CRI improvement from this strategy is generally smaller than that from internal edge addition, it still contributes positively to overall network integrity.

Both heuristics improve their designated metrics in the tested networks, but the gains are conditional on the selected attack and budget. Intra-cluster edge addition is more suitable when the target is internal-efficiency retention under centrality-based removal, whereas cross-cluster rewiring is more suitable when the target is external-efficiency retention under bridge-node removal. For the World Trade topology, the larger relative gains indicate more room for these two topological interventions under the adopted budgets; they do not establish an operational or economic effect.

## 4. Conclusions

This study separates internal and external cluster efficiency and evaluates their retention under node removal. The revised definitions use the mean reciprocal shortest-path distance, resolving the inconsistency between a harmonic efficiency concept and the reciprocal of an arithmetic mean distance. CRI records combined structural retention, SRE describes component-size fragmentation, and AUC–*I* and AUC–*E* summarize retention of the two cluster-efficiency components.

Across the tested BA, LFR, and empirical topologies, targeted attacks based on degree, betweenness, k-shell, or bridge information cause larger losses than random failures. Small clusters and clusters dominated by a few central nodes are generally more sensitive, while distributed internal connectivity and diversified external links improve retention. The complete formula audit shows that using $\bar{d^{-1}}$ instead of $1/\bar{d}$ is essential for consistency with harmonic network efficiency, but it leaves most normalized curve shapes and attack rankings nearly unchanged; the largest visible differences are concentrated in the weighted-average efficiency results. This limited numerical effect is itself an important finding because it separates correction of the metric definition from the stability of the broader robustness conclusions. The intra-cluster addition heuristic mainly improves AUC–*I* and CRI, whereas cross-cluster rewiring mainly improves AUC–*E* under bridge-oriented attack. These findings are conditional on the unweighted, static graphs, detected Louvain partitions, attack definitions, and budgets used here; they should not be interpreted as direct evidence about economic productivity, collaboration quality, traffic volume, transmission speed, or post-failure recovery.

Future work should test weighted and temporal networks, compare alternative community-detection uncertainty, incorporate explicit flow or dynamical models, and optimize under realistic edge costs and degree-preservation constraints. Releasing the revised Python implementation, parameter manifest, and unit tests is intended to make the metric definitions and synthetic experiments auditable; reproducing the five empirical figures additionally requires the original edge-list data described in the code package.

## Figures and Tables

**Table 1:** Comparison of average cluster efficiency vulnerabilities in LFR and BA networks.

Network	$V_{\mathrm{internal}/\mathrm{external}}$ of Large Clusters	$V_{\mathrm{internal}/\mathrm{external}}$ of Small Clusters
BA	0.22/0.04	0.31/0.11
LFR	0.24/0.08	0.45/0.12

**Table 2:** The index change of the LFR network under two robust optimization strategies.

Index	Pre-Optimization	Optimization (Edge Addition)	Optimization (Edge Rewiring)	Enhancement Range
AUC-I	0.0710	0.0728	–	2.54%
AUC-E	0.0811	–	0.0828	2.10%
AUC-CRI	0.2438/0.3566	0.3654	0.6000	19.88%/2.45%

**Table 3:** Real-world networks for evaluation.

Network	*N*	*E*	<k>	$k_{\max}$	$n_{L}$	$n_{L}/N$	$n_{S}$	$n_{S}/N$	$n_{L}/n_{S}$
Polbooks	105	441	8.4	25	44	0.419	8	0.076	5.5
Netscience	379	914	4.8	34	43	0.113	6	0.016	7.2
Yeast	1458	1948	2.6	56	84	0.058	13	0.009	6.5
MU	186	1715	18.4	125	53	0.285	15	0.081	3.5
World Trade	244	5402	44.3	238	120	0.492	16	0.066	7.5

**Table 4:** Improvement in robustness indices across five real-world networks under two robustness optimization strategies.

Network	AUC−I	AUC−E	AUC−CRI (Edge Addition)	AUC−CRI (Edge Rewiring)
Polbooks	2.42%	2.45%	11.9%	1.12%
Netscience	1.07%	0.04%	5.62%	0.44%
Yeast	1.63%	0.44%	8.09%	1.39%
MU	3.47%	1.92%	7.28%	2.27%
World Trade	4.13%	2.19%	8.55%	2.33%

## Data Availability

The revised Python implementation, parameter manifest, and unit tests accompany this revision and reproduce the metric definitions and synthetic-network workflow. The BA and LFR experiments can be regenerated from the recorded random seeds and settings. The empirical analyses use the edge-list topologies described in Section 3; full independent regeneration of those figures requires the corresponding original edge-list files and their source-specific licenses. No synthetic replacement data were used for the empirical results.

## Appendix A

**Table A1:** Consolidated table of notations.

Parameters	Definition
*N*	Total number of network nodes
*E*	Total number of network edges
<k>	Average degree of the network
$k_{max}$	Maximum degree of a network node
$n_{L}$	Number of nodes in the largest network cluster
$n_{S}$	Number of nodes in the smallest network cluster
